# The effect of local hospital waiting times on GP referrals for suspected cancer

**DOI:** 10.1371/journal.pone.0294061

**Published:** 2024-05-08

**Authors:** Helen Hayes, Rachel Meacock, Jonathan Stokes, Matt Sutton

**Affiliations:** 1 Office of Health Economics (OHE), London, United Kingdom; 2 Health Organisation, Policy and Economics (HOPE), Centre for Primary Care & Health Services Research, School of Health Sciences, The University of Manchester, Manchester, United Kingdom; 3 MRC/CSO Social & Public Health Sciences Unit, School of Health and Wellbeing, University of Glasgow, Glasgow, United Kingdom; 4 Centre for Health Economics, Monash University, Melbourne, Victoria, Australia; University of Porto, Faculty of Medicine, PORTUGAL

## Abstract

**Introduction:**

Reducing waiting times is a major policy objective in publicly-funded healthcare systems. However, reductions in waiting times can produce a demand response, which may offset increases in capacity. Early detection and diagnosis of cancer is a policy focus in many OECD countries, but prolonged waiting periods for specialist confirmation of diagnosis could impede this goal. We examine whether urgent GP referrals for suspected cancer patients are responsive to local hospital waiting times.

**Method:**

We used annual counts of referrals from all 6,667 general practices to all 185 hospital Trusts in England between April 2012 and March 2018. Using a practice-level measure of local hospital waiting times based on breaches of the two-week maximum waiting time target, we examined the relationship between waiting times and urgent GP referrals for suspected cancer. To identify whether the relationship is driven by differences between practices or changes over time, we estimated three regression models: pooled linear regression, a between-practice estimator, and a within-practice estimator.

**Results:**

Ten percent higher rates of patients breaching the two-week wait target in local hospitals were associated with higher volumes of referrals in the pooled linear model (4.4%; CI 2.4% to 6.4%) and the between-practice estimator (12.0%; CI 5.5% to 18.5%). The relationship was not statistically significant using the within-practice estimator (1.0%; CI -0.4% to 2.5%).

**Conclusion:**

The positive association between local hospital waiting times and GP demand for specialist diagnosis was caused by practices with higher levels of referrals facing longer local waiting times. Temporal changes in waiting times faced by individual practices were not related to changes in their referral volumes. GP referrals for diagnostic cancer services were not found to respond to waiting times in the short-term. In this setting, it may therefore be possible to reduce waiting times by increasing supply without consequently increasing demand.

## Introduction

Waiting times are a major policy concern in many OECD countries and many strategies have been implemented to reduce the length of time that patients wait for health care [1]. Patients experience waits at multiple points along the care pathway, first waiting for an initial appointment and then again for diagnostic results, before furthermore waiting between receipt of diagnosis and commencement of treatment. Prolonged waiting times can lead to inconvenience, discomfort and dissatisfaction for patients. In some cases, longer waits can lead to worsening health during the waiting period, cancers being diagnosed later, or a reduction in the health gain from treatment when it is finally received [2–4]. Nevertheless, waiting times are a common feature of publicly funded health systems with capacity constraints, where they operate as a rationing device in the absence of prices.

Waiting times can affect the demand for healthcare services. Patients may be deterred from treatment if waiting times are excessive, or they may seek private treatment instead [2]. Healthcare professionals play a key role in the demand for healthcare in many health systems. In the UK for example, general practitioners (GPs) act as gatekeepers for patients, who must receive a referral from a GP in order to access appointments for specialist hospital services.

Previous economic studies have assessed how responsive healthcare demand is to changes in waiting times, termed ‘demand elasticity’. A highly elastic response is one where a small change in waiting times leads to a large change in healthcare demand, whereas an inelastic response is one where a change in the demand is relatively unaffected by a change in waiting times. The current evidence finds the elasticity of demand for healthcare with respect to waiting times to be negative, and generally small [5–9]. This means that demand rises as waiting times fall, but this increase is less than proportionate to the change in waiting times (i.e., relatively inelastic). These findings suggest that increasing supply can significantly improve waiting times, without being offset substantially by subsequent increases in demand.

Much of the current waiting times literature explores demand responses in elective care. There is very limited evidence on demand responses to waiting times in more urgent contexts. One study found that when waiting times in emergency departments were higher, there was reduced demand from low-urgency patients but the elasticity of demand for these patients was relatively low [10]. This suggests that the demand response in an urgent care facility may be in line with that for elective care when urgency is low.

There is a particular lack of evidence on the GP demand response to waiting times for urgent but non-emergency care, such as diagnosing a condition in need of rapid care. There is greater uncertainty over a patient’s clinical need in situations where patients are being referred for diagnosis, as opposed to referral for treatment. The time cost associated with waiting is also more complex in a diagnostic setting, as it is dependent on both (1) whether the patient has the condition or not and (2) how serious the condition is, neither of which are known prior to diagnosis.

Cancer diagnosed at an early stage is associated with reduced costs of treatment and a higher chance of survival [11, 12], making it a relevant disease to explore the GP demand response to waiting times, as there is a high time cost to waiting for a diagnosis. Furthermore, the COVID-19 pandemic has put a strain on cancer services and caused increased delays to diagnosis and delivering treatment [13]. There is a growing backlog of secondary care that the NHS was unable to deliver due to the pandemic. In the context of cancer services this includes patients who were unable to be diagnosed or treated. There will be increased demand and further pressure on services in the future, as patients who were unable to receive treatment find their conditions have worsened, and those who went undiagnosed will present to services at a later stage [14].

This paper examines the effect of local hospital waiting times for urgent suspected cancer initial appointments on GP demand for urgent referrals, using nationally-representative administrative data in England. It adds to the literature on the demand response of GPs to waiting times in the under-researched area of urgent but non-emergency care. The results of this study are particularly important as long waiting times and waiting lists for cancer services caused by the COVID-19 pandemic are likely to persist in future years.

## Background

### The role of general practitioners in the urgent referral process in England

In England, patients are entitled to NHS healthcare which is funded through general taxation and generally free at the point of use. Patients in England register with a single GP practice, which is then responsible for the patient’s ongoing medical care, across care settings. Patients can access appointments for routine and urgent primary care through their GP practice. Patients can directly use hospital services in an emergency, but GPs act as gatekeepers for non-emergency hospital services which require a referral.

For cases of suspected cancer, a GP can request an urgent referral for the patient to see a specialist hospital doctor. Urgent referrals can be made for patients of any age, but NICE guidance on whether to refer will vary by age group. This is named a ‘two-week wait’ referral, as a waiting time target introduced in 2000 states that this specialist appointment should take place within two weeks of referral [15]. For this study, we refer to this type of referral as an urgent referral. Referrals via the two-week wait route are the most common route to a cancer diagnosis. Of the eight main routes to a cancer diagnosis in England, 38% of all cancer diagnoses came via the two-week wait route in 2016 [16]. S1 Appendix contains details of the full list of routes to a cancer diagnosis in England.

### The GP decision problem

The GP’s decision to refer a patient is influenced by a complex set of potentially competing factors in addition to what they are able to observe about the patient’s clinical need. The GP needs to weigh up the benefit of a referral for a patient (e.g., receiving specialist care that the GP cannot provide; patient desire for a referral) with the potential costs of a referral to the patient (e.g., the time and effort of going to see the specialist and the anxiety over waiting for a separate referral) and to the health system and therefore other patients (i.e. opportunity costs). Increased waiting times may exacerbate these potential costs, and thus result in a decrease in the patient’s net benefit from referral, which could impact a GP’s decision to refer, or affect referral destination [17].

The decision is further impacted by GP factors such as risk averseness of the GP. At an individual-level, it is reasonable to assume that more risk averse GPs are more likely to make more referrals to secondary care [18]. Other structural factors may also impact a GP’s decision to refer, such as how GPs are paid and market competition [18, 19]. All of these influences contribute to a GP’s ‘referral threshold’, which may vary widely between GPs, and over time as systems change.

#### The role of waiting times in GP referral decisions for suspected cancer

Whilst NICE guidelines provide recommendations on symptoms which warrant an urgent referral for suspected cancer, signs such as weight loss and fatigue are not always distinguishable from other less serious conditions. GPs must therefore find a balance between referring all patients and inducing unnecessary anxiety and potential overtreatment through further testing [20]. GPs may have different thresholds for referring to hospital when symptoms are unclear, and this may impact outcomes [21]. Thresholds for cancer referral have been found to vary significantly between general practices despite the presence of these guidelines, as has the accuracy of referral decisions [22]. Research has also found compliance with the NICE referral guidelines to be poor even in situations where patients present to their GP with “red flag” features including breast lumps and rectal bleeding [23]. Most patients subsequently diagnosed with cancer see their GP multiple times before being referred to specialist hospital services [24, 25], providing further evidence that accurately spotting cancer is difficult even with the aid of referral guidelines.

We hypothesise that whilst GPs will always refer patients whom they suspect to have cancer, local hospital waiting times may influence referral decisions on the margin for patients whom the GP believes cancer is unlikely, i.e., for patients where the diagnosis is particularly unclear. The direction of the effect could therefore go either way, driven by two competing possible response mechanisms.

Amongst patients for whom the GP does not strongly suspect cancer but an alternative diagnosis is also not obvious, reduced waiting times on the suspected cancer pathway may lead GPs to refer more patients with the hope of reducing the period of uncertainty for the patient and ruling out cancer. This would mirror previous findings on the GP demand response to shorter waiting times for non-urgent and elective surgery [26, 27], where referral volumes were reported to rise as waiting times fell.

Alternatively, shorter waiting times on the suspected cancer pathway may act as a safety net for GPs who do not suspect cancer, allowing them more scope to explore other alternatives first in the knowledge that subsequent waiting times will be short if these explorations do not result in an alternative diagnosis. Anxiety around cancer is very high compared to other conditions [28], and so GPs may prefer not to raise the possibility of cancer with patients unnecessarily when they believe it to be unlikely. However, when waiting times are long, GPs may decide it is necessary to start the referral process early just in case, and so be more inclined to refer patients whom they do not believe have cancer onto this pathway as a precaution.

A priori, we do not know which of these potential mechanisms will dominate.

#### Receipt of waiting times information by GPs

Waiting times can only play a role in referral decisions if GPs have access to information on waiting times at the local hospitals to which they can refer. This information would have reached GPs in at least two ways during the period we study. Hospital performance against the national waiting times standards was publicly reported on a monthly basis [29], with a three month delay. GPs were therefore able to access this performance data easily, and could follow trends over time at each hospital. Secondly, GPs could observe the waiting times of their own patients directly once they were referred. The average general practice in England made the equivalent of approximately 65 annual referrals for suspected cancer per full time equivalent GP [30]. GPs therefore repeatedly observed the waiting times experienced by their own patients throughout the year, providing direct personal experience of whether these were changing over time.

## Methods

We modelled the GP demand response to waiting times using GP practice level data on urgent referral volumes for suspected cancer patients and local hospital waiting times. This method assumes that GP practices are ‘price-takers’ in terms of the waiting times they experience, meaning that they must accept the waiting times they face from hospitals and each individual practice has no control over these waiting times. We assumed this to be the case as many practices refer to each hospital, meaning that the number of patients that a single practice refers has little influence on aggregate hospital waiting times. We assessed the plausibility of this assumption in our analysis. We also assume that demand responds contemporaneously to waiting times for a suspected cancer referral, given that we used annual data (as e.g. [5]).

### Data

#### Sample

Our sample consisted of 6,667 GP practices referring to 185 hospitals in England, between the financial years 2012/13 and 2017/18. We obtained annual data on GP practices from the 2018/19 update of Public Health Profiles provided by Public Health England [31]. Only practices with a list size of over 1,000 registered patients in 2018/19, and practices which are in the GP Patient Survey (a patient experience survey of services provided by the GP practice) are included in the Public Health Profiles data.

We obtained hospital level data on waiting times from NHS England [32]. We used Hospital Episode Statistics (HES) data on two-week wait appointments to link practices to local hospitals. As data from HES are available up to the financial year 2017/18 we included up to this year of GP practice level data from Public Health Profiles in our analysis. The final sample included 37,556 practice-year observations. We do not have data for all periods for all practices due to practice openings and closures during the period of interest. There were also 8,208 missing observations on practices when they did not refer any two-week wait patients in HES in a given year. We carried out a sensitivity analysis restricting the analysis to practices with observations in every year.

Ethical approval was not required for this study as data obtained were publicly available, published data on English GP practices and hospitals. HES data received for the purpose of linking the practice and hospital level data had been de-identified.

#### Variables

*Outcome variable*. We examined the practice-level count of urgent GP referrals for suspected cancer. Urgent GP referrals were assigned to the financial year in which that patient was referred. It is possible that an individual patient could have appeared more than once, but only if they were referred for suspected cancer on multiple occasions by their GP.

*Variable of interest*. Our variable of interest was local hospital waiting times. Average length of waiting times for suspected cancer are not published at the practice or provider level. Therefore, as a measure of waiting times performance, we used hospital breaches of the two-week wait target, as a proportion of total suspected cancer patients referred [32]. This measure was calculated retrospectively, based on how long patients who were seen for an urgent referral had waited since the time of referral.

Waiting time breaches as a proportion of total patients referred are available at the hospital level. Many practices do not exclusively refer all of their patients to a single hospital, although there are some that do. For each general practice we therefore created a weighted local hospital waiting times measure. The measure was weighted by the observed shares of urgent suspected cancer referrals from the practice to each hospital taken from HES. We used the average shares over time for each practice to avoid changes in hospital referral destination as a potential response to waiting times.

The calculation of the local waiting times indicator (*LW*_*pt*_) is outlined in Eq 1, such that:

$${LW}_{pt}=\sum_{h=1}^{n}W_{ht}S_{ph}$$ Eq 1

In which,

*p* = practice

*h* = hospital

*n* = number of hospitals to which the practice refers suspected cancer patients

*W*_*ht*_ = breaches of the two week wait rule as a proportion of the total suspected cancer patients referred, in a given hospital-year

$$S_{ph}=\frac{{R^{HES}}_{ph}}{{R^{HES}}_{p}}$$

*R*^*HES*^_*ph*_ = total (attended) referrals from practice *p* to hospital *h*

*R*^*HES*^_*p*_ = total (attended) referrals made by practice *p*

$$\sum_{h=1}^{n}S_{ph}=1$$

0<S≤1

*Covariates*. We included several covariates which may be correlated with local hospital waiting times and GP referral activity. We controlled for: the size of the population registered with the GP practice (list size); characteristics of the registered practice population’s age (proportion aged under 18, proportion aged 65+), health status (proportion with a long-standing health condition) and employment status (proportion unemployed). We also controlled for patient-reported access to the practice from the GP Patient Survey (proportion reporting good experience of making an appointment, proportion satisfied with phone access), and clinical quality (proportion Quality and Outcomes Framework (QOF) points achieved across all domains as a proportion of all achievable points). The QOF measures practice performance against a number of quality indicators [33].

### Empirical strategy

#### GP demand analysis

We explored a general model of the form expressed in Eq 2:

$${R^{PHP}}_{pt}=f({LW}_{pt},X_{pt},\tau_{t},\varepsilon_{pt})$$ Eq 2

Where *R*^*PHP*^_*pt*_ is total referrals (to any hospital) from GP practice *p* in year *t*. The PHP superscript indicates that this practice-level measure of two-week wait referrals was taken from the Public Health Profiles data (Public Health England, 2020). *LW*_*pt*_ is the local average waiting time faced by practice *p* in year *t* (see the section “Variable of interest” for calculation). ***X***_***pt***_ are the set of practice-level, time-varying covariates outlined in the section “Covariates”. *τ*_*t*_ denotes time fixed effects and *ε*_*pt*_ is the idiosyncratic error term.

We first estimated Eq 3 using pooled OLS:

$${R^{PHP}}_{pt}=\beta_{0}+\beta_{1}{LW}_{pt}+\beta_{2}X_{pt}+\tau_{t}+\varepsilon_{pt}$$ Eq 3

Next, we estimated between-practice effects in Eq 4 using OLS. This focuses on the cross-sectional association between waiting times and referrals by regressing the mean of the outcome variable, across all time periods, for each practice, on the mean values of each of the explanatory variables:

$${\bar{R^{PHP}}}_{p}=\beta_{0}+\beta_{1}{\bar{LW}}_{p}+\beta_{2}{\bar{X}}_{p}+\varepsilon_{p}$$ Eq 4

We then estimated the within-practice effects using a Dummy Variable Ordinary Least Squares model:

$${R^{PHP}}_{pt}=\beta_{0}+\beta_{1}{LW}_{pt}+\beta_{2}X_{pt}+\alpha_{p}+\tau_{t}+\varepsilon_{pt}$$ Eq 5

Eq 5 is an altered version of Eq 3 which now includes fixed effects (or dummy variables) for each practice (*α*_*p*_). These control for factors that are not measured but are constant over time for each practice (such as location).

By utilising these three regression models, we can identify whether associations we find are driven by between-practice (cross-sectional) or within-practice (over time) variation. As the fixed effects analysis controls for unobserved characteristics of a practice which are constant over time, this is the analysis least vulnerable to unobserved confounding.

We used inverse hyperbolic sine transformations of all variables in all models. Using inverse hyperbolic sine transformations is recommended to transform right-skewed variables which include zeros, approximating a normal distribution and reducing the effect of outliers [34]. The inverse hyperbolic sine transformation of a variable approximates the natural logarithm of that variable, while not losing observations which are zero. Transforming these variables allowed us to interpret the coefficients as elasticities. In this instance, these will measure the elasticity of demand with respect to waiting times, i.e. the responsiveness of GP referral demand to changes in hospital waiting times. An elasticity estimate of -1 would indicate that a one percent increase in waiting times breaches would lead to a one percent reduction in referral volumes. An elasticity estimate of between -1 and 0 would indicate that GP demand is inelastic with respect to waiting times, as a one percent increase in waiting times breaches would lead to a reduction in referrals of less than one percent.

#### Robustness checks

*Testing the assumption that GP practices are price-takers*. A key assumption in our analysis is that a single GP practice cannot influence local hospital waiting times (i.e. that GP practices are ‘price-takers’ in terms of the local waiting times they experience). To confirm the validity of this assumption we created an index which measures the degree to which this assumption is held, which we call the ‘concentration of referrals index’.

This index measures the concentration of referrals going from a practice to any single hospital, weighted by the concentration of referrals that a hospital receives from that practice. The proportion of a hospital’s referrals that come from a single practice will reflect the degree to which a practice is a price-taker for that particular hospital, as a practice is less likely to influence the hospital’s waiting times if it represents a lower proportion of its activity. However, as we are concerned with whether the practice is a price taker for the overall local waiting time it experiences, we also need to consider whether the referrals that a particular practice makes to a particular hospital are also a large proportion of the practice’s total referral volumes.

For each practice, in each year, we calculated the concentration of referrals index as outlined in Eq 6 using data from HES. For each practice-year, we multiplied the share of a hospital’s referrals which come from that practice in that year with the share of a practice’s referrals going to that hospital. We repeated this for each hospital and created the concentration of referrals index as the sum of these values in each practice-year:

$${CRI}_{pt}=\sum_{h=1}^{n}\frac{R_{pht}}{R_{ht}}*\frac{R_{pht}}{R_{pt}}$$ Eq 6

The index will equal 1 if a practice sends all of its activity to a hospital where it is the only referrer in that year. More generally, the higher the concentration of referrals index, the more influence the practice has over its local hospital waiting times in that year.

We then examined the average concentration of referrals index for each practice across all years in our sample, to evaluate whether the price-taker assumption holds for each practice in general.


$${CRI}_{p}=\sum_{h=1}^{n}\frac{R_{ph}}{R_{h}}x\frac{R_{ph}}{R_{p}}$$ Eq 7

*Further sensitivity checks on the GP demand analysis*. We repeated estimation of Eqs 3–5 using a Poisson regression on the untransformed data, to allow for the count nature of the outcome variable. For the Poisson regressions, we used the GP practice’s registered list size as the exposure variable.

We then examined whether the effect we find persists when we modelled the outcome as a rate. To do this we repeated the estimation of Eqs 3–5, where the outcome variable is the referral rate (urgent referrals as a proportion of the practice population), and population size is not included as a covariate.

We also repeated the main analysis (Eqs 3–7) on a balanced panel, removing practices which do not appear in all six years of data.

#### Supplementary outcomes analysis

Finally, we conducted some further analysis to examine the validity of the local waiting times measure. Our concern over the local waiting times measure is twofold: (1) we used waiting time breaches as a proxy for actual waiting times, and (2) our calculation which used weighted values of local hospital waiting times may not accurately reflect average waiting times for the specific patients of each practice.

To check that our measure of local waiting times was valid, we also examined the association between our measure of local hospital waiting times and patient outcomes. If there was an association between waiting times and practice-level patient outcomes, this provides reassurance that our measure of local hospital waiting times is valid.

Specifically, we hypothesise that longer waiting times will result in an increase in diagnoses via emergency presentation. Emergency presentations of cancer tend to be later stage and result in worse outcomes [35]. We therefore proxied patient outcomes by comparing the number of diagnoses via urgent referral (the appropriate pathway) and diagnoses via emergency presentation. These are two of eight possible routes to diagnosis (see S1 Appendix). We carried out fixed effects analysis following Eq 5, but using these two variables (urgent referral diagnoses and diagnoses via emergency presentation) as outcomes. To control for the number of diagnoses via any route, we included the inverse hyperbolic sine transformed count of total cancer diagnoses at the practice level as a covariate.

## Results

### Descriptive statistics

Summary statistics on the key variables in our analysis are presented in Table 1. Average practice list size was 7,893 registered patients. On average, a GP practice urgently referred 220 suspected cancer patients (excluding patients who are not followed up with a specialist appointment) in each financial year in our period of analysis. The weighted local hospital waiting time breaches measure was on average 5.2%, corresponding to the average proportion of patients referred via the urgent referral pathway not being seen by a specialist within two weeks.

**Table 1:** Summary statistics on key variables at the GP practice level, 2012/13-2017/18.

	Mean	SD	Min	Max	Obs
*GP demand*					
Count of urgent referrals	220.501	165.601	0.000	2183.000	37556
*Waiting times*					
Target breaches as proportion of referrals	0.052	0.023	0.000	0.225	37556
*Covariates*					
Registered population size	7893.013	4632.028	1001.000	72227.000	37556
Proportion aged 65+ years	0.168	0.066	0.000	0.952	37556
Proportion aged under 18 years	0.209	0.042	0.000	0.539	37556
Total QOF points achieved (proportion)	0.959	0.059	0.161	1.000	37556
Working status—Unemployed	0.055	0.050	0.000	0.654	37556
Proportion reporting good overall experience of making appointment	0.743	0.136	0.111	1.000	37556
Proportion satisfied with phone access	0.753	0.175	0.074	1.000	37556
Proportion with a long-standing health condition	0.531	0.081	0.083	1.000	37556

Table 2 shows that local hospital waiting times have varied over time, with waiting times worsening after 2013/14. There was variation in the number of practices included in the dataset over time due to lack of information on a practice’s urgent suspected cancer referrals from HES. There was further variation as Public Health Profiles data includes practices based on which practices are open in the most recent year of data, hence why the sample size was greater in later years of the sample. In comparison with the sample size over time in our sample, the number of practices have decreased in recent years due to practice closures or mergers [36].

**Table 2:** Average annual values of the practice-level local hospital waiting times measure, at the GP practice level.

	2012/13	2013/14	2014/15	2015/16	2016/17	2017/18
Local hospital breaches as a proportion of total patients referred	0.045	0.045	0.057	0.057	0.052	0.055
Observations	6026	6287	5788	6372	6573	6510

**Fig 1:**
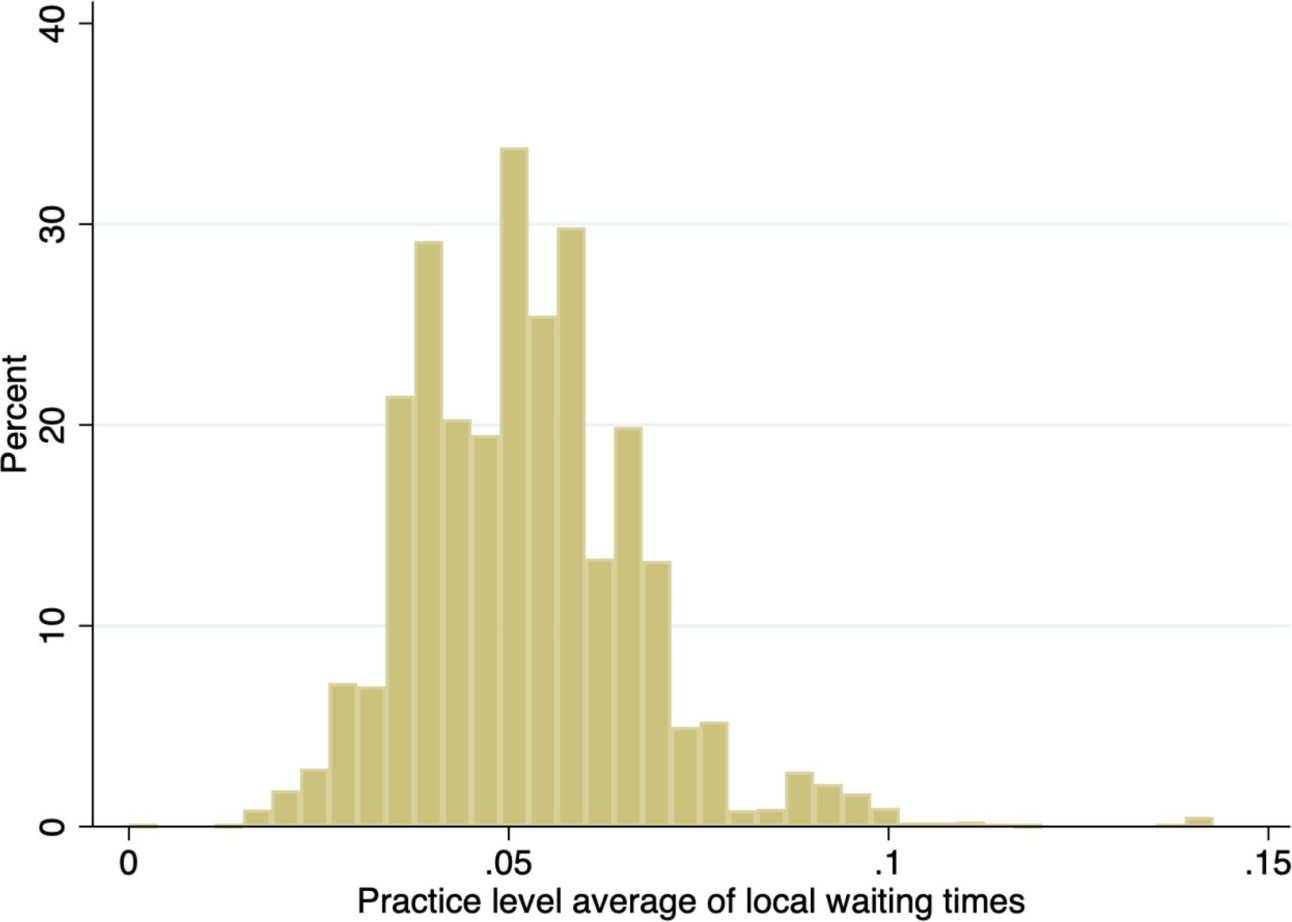
Histogram of practice-level averages of local waiting time breaches as a proportion of total referred.

Fig 1 shows practice level values of the local waiting times measure, averaged across all years. This figure illustrates the variation in the local waiting times faced by general practices, with average breaches at local hospitals ranging from 0% to 14%.

Table 3 decomposes the overall variation in the local waiting times measure into the variation between practices and the variation within practices over time. These figures show that there is more variation within practices over time than there is between practices. Together, Fig 1 and Table 3 demonstrate that in our sample there is sufficient variation in the measure of local waiting times to facilitate between- and within-practice analyses.

**Table 3:** Between and within practice variation statistics.

Variable		Mean	SD	Min	Max	Obs
Local hospital breaches as a proportion of total patients referred	Overall	0.052	0.023	0.000	0.225	N = 37556
	Between		0.015	0.000	0.143	n = 6667
	Within		0.0175	-0.020	0.187	T = 5.633

### GP demand analysis

In the pooled OLS model and the between-practice estimator, there was a positive correlation between waiting times and GP demand for urgent referrals: a 10% increase in local hospital breaches of the two-week wait target was associated with higher referral volumes by 4.4% (p<0.01) and 12.0% (p<0.01), respectively (Table 4). However, the within-practice estimator did not reveal a statistically significant relationship (1.0%, p>0.1).

**Table 4:** Relationship between GP demand and local hospital waiting times.

	Volume of urgent referrals		
	Pooled	Between effects	Fixed effects
Target breaches as proportion of referrals	0.440***	1.202***	0.100
	(0.104)	(0.331)	(0.0742)
Proportion aged 65+ years	1.201***	0.751***	-1.145***
	(0.0558)	(0.137)	(0.255)
Proportion aged under 18 years	-0.257***	-0.174	-0.651**
	(0.0791)	(0.145)	(0.255)
Total QOF points achieved (proportion)	0.231***	0.251*	0.0860*
	(0.0538)	(0.148)	(0.0458)
Proportion unemployed	-0.309***	-0.835***	-0.0567
	(0.0638)	(0.170)	(0.0412)
Proportion reporting good overall experience of making appointment	0.177***	0.287**	-0.0139
	(0.0399)	(0.115)	(0.0314)
Proportion with a long-standing health condition	0.379***	0.917***	-0.0384
	(0.0405)	(0.125)	(0.0241)
Proportion satisfied with phone access	-0.172***	-0.227***	0.0189
	(0.0298)	(0.0827)	(0.0290)
Practice list size	1.160***	1.162***	0.864***
	(0.00480)	(0.00878)	(0.0230)
Adjusted R^2^	0.735	0.755	0.579
N*T	37556	6,667	37556
GP practice fixed effects	NO	NO	YES
Robust standard errors	YES	NO	YES
Year fixed effects	YES	NO	YES

Notes: all variables are inverse hyperbolic sine transformed.

Standard errors in parentheses.

* p<0.10

** p<0.05

*** p<0.01.

As the pooled estimate is a weighted average of the estimates from the between and within models, this suggests that the pooled effect is largely driven by the variation across practices. This indicates that other factors at the GP practice level which we were unable to control for were creating the association between local hospital waiting times and GP demand in the pooled analysis.

Once we adjust for unmeasured characteristics of practices that are constant over time (such as location) and focus on the correlation between changes in waiting times experienced by practices and changes in their referrals, this effect greatly reduced in magnitude and was no longer statistically significant.

### Sensitivity checks

#### Testing the assumption that GP practices are price takers

Table 5 provides descriptive statistics on the concentration of referrals indices for each practice-year (N*T values), and for each practice on average across all years (N average values). There were a few cases where low numbers of referrals between practice and hospital in a given year create a high concentration of referrals index in a practice-year. For example, the maximum value of 1 (row 1, Table 4) was due to a single practice making only one referral to a hospital which only received one urgent referral in that year. Averaging over all years for each practice (row 2, Table 4) the mean concentration of referrals index across all practices was 0.014. We can therefore conclude that practices did not consistently dominate a hospital’s referrals and were not dominated by certain local hospitals over our period of interest.

**Table 5:** Summary statistics on the indices of concentration of referrals between practices and hospitals.

Variable	Obs	Mean	Std. dev.	Min	Max
1. All practice-year indices	37,556	0.0140614	0.0162474	0.0000181	1
2. Practice average indices	6,667	0.0140806	0.0142864	0.0000403	0.25

#### Further sensitivity checks on the GP demand analysis

Sensitivity analyses of the GP demand analysis using Poisson regressions, and where the outcome variable is modelled as the referral rate as a proportion of the practice population (S2 and S3 Appendices) supported the main analysis.

Both sets of results exhibited the same pattern as the main analysis. There was a statistically significant positive association between local hospital waiting times and GP referrals for pooled OLS and the between estimator, and a smaller and statistically insignificant positive association for the fixed effects analyses.

Repeating the analysis using a balanced panel supports the main results in terms of the sign, significance and magnitude of the associations (S4 Appendix). Unadjusted estimates are presented in S5 Appendix.

### Supplementary outcomes analysis

Summary statistics for the outcomes analysis are presented in S6 Appendix. On average, 17.8 patients registered with a GP practice were diagnosed via urgent referral each year during our analysis period, whilst 7.0 patients were diagnosed via emergency presentation.

Table 6 shows that, as expected, longer hospital waiting times were associated with worse patient outcomes in terms of route of diagnosis. We found that higher local hospital waiting times breaches were associated with a greater number of cancer patients being diagnosed via emergency presentation, and a lower number being diagnosed by the preferred two-week wait urgent referral pathway. A 10% increase in local hospital breaches of the two-week wait target was associated with a -2.7% reduction in cancers diagnosed via the appropriate route of two-week wait urgent referral and a 2.4% increase in cancers diagnosed via emergency presentation.

**Table 6:** Inverse hyperbolic sine transformed linear relationship between patient outcomes and local hospital waiting times.

	Diagnoses via urgent referral	Diagnoses via emergency presentation
Local hospital breaches as a proportion of total treated	-0.266**	0.241*
	(0.120)	(0.146)
Proportion aged 65+ years	-0.816**	1.317***
	(0.391)	(0.447)
Proportion aged under 18 years	-0.791**	0.555
	(0.391)	(0.473)
Total QOF points achieved (proportion)	0.0532	-0.192**
	(0.0774)	(0.0951)
Proportion unemployed	0.0286	0.0917
	(0.0783)	(0.0932)
Proportion reporting good overall experience of making appointment	-0.0701	-0.0967
	(0.0520)	(0.0607)
Proportion with a long-standing health condition	-0.0372	-0.0874
	(0.0437)	(0.0549)
Proportion satisfied with phone access	0.0850*	-0.0344
	(0.0468)	(0.0544)
Total cancer diagnoses	0.0440***	0.677***
	(0.0105)	(0.0138)
Adjusted R^2^	0.0986	0.112
N*T	37517	37517
Robust standard errors	YES	YES
GP practice fixed effects	YES	YES
Year fixed effects	YES	YES

Notes: all variables are inverse hyperbolic sine transformed.

Standard errors in parentheses.

* p<0.10

** p<0.05

*** p<0.01.

These analyses indicate that patient outcomes were sensitive to the waiting time measure we used and increases confidence in the appropriateness of the within-practice estimator for GP demand.

## Discussion

We examined the relationship between a GP practice’s local hospital waiting times and the GP demand for urgent referrals of patients with suspected cancer. Our analysis revealed a positive association between waiting times at local hospitals and the demand for urgent suspected cancer diagnostic services by GPs, but this correlation was driven by cross-sectional differences between practices. Practices with higher levels of referrals experienced longer waiting times at local hospitals but these are likely both due to unmeasured local population or care system factors.

When we focused on within-practice variation using a fixed-effects model, we found that changes in the waiting times experienced by practices did not have a significant effect on their volume of referrals. These results suggest that GP referral behaviour was not responsive to changes in waiting times in the short-term. This suggests that increases in supply in this setting could have significant impacts on waiting times without affecting demand, at least in the short run.

Our supplementary analysis suggests that there was a negative association between changes in local waiting times and changes in patient outcomes at the practice level. This provides reassurance both that the fixed-effects model was appropriate and that the indicator of local waiting times we used in this study was sufficiently sensitive.

### Strengths and limitations of the study

This study adds to the literature on the demand response of GPs to waiting times for a cancer diagnosis, at a time when the NHS is struggling to meet its targets for cancer waiting times, exacerbated by the COVID-19 pandemic. We used a nationally-representative dataset on GP practices and their local hospitals in England to answer this question.

Other studies which have looked to estimate the elasticity of demand with respect to waiting times have used measures of activity which reflect both supply and demand, such as the number of treatments delivered [5, 6, 37]. For this to represent the elasticity of demand it is necessary to assume that supply and demand are in equilibrium. We used the volume of urgent referrals made by GPs as our measure of activity, which directly reflects their demand for these specialist services.

In this analysis, we used a proxy measure for waiting times. The two main issues with this measure are that firstly, it is the percentage of breaches and not the actual waiting times, and secondly it is the average across local hospitals. There may be cases where the waiting times experienced by a specific practice’s patients differs from the local hospital average. However, the supplementary patient outcomes analysis suggests that outcomes at a practice-level were sensitive to the measure of local hospital waiting times that we used.

As activity is measured at the GP practice level, we assume any GP practice’s referrals cannot have a significant impact on the waiting times at any given hospital. This assumption that GPs are price takers alleviates the concern that waiting times and demand are associated because of reverse causality [8]. Our analysis on the concentration of referrals supports this assumption. However, there is still some concern that the assumption that GP referrals cannot affect waiting times may not hold in practice. For example, an unmeasured local demand shock would increase referrals, and if the supply response is not sufficient, more patients might therefore breach.

The fixed effects analysis controls for unmeasured factors which vary across practices and are constant over time for each practice, such as location. However, it is unable to mitigate bias caused by practice-level time-varying unmeasured factors which are correlated with both local hospital waiting times and GP urgent referrals. Such factors, which we are unable to control for due to lack of data, could include screening uptake in the area, variability in the ability to diagnose suspected cancer patients within the practice, or changes in the likelihood of using other routes to diagnosis than the two-week wait pathway. This means that our result could be an under or over-estimate of the true effect, depending on the direction of the relationship between the omitted variables, waiting times and GP referrals. In addition, because we do not have individual patient data, we cannot adjust for patient-specific factors such as travel distance to the practice and hospital.

Cancer is not just one disease. There are over 200 types of cancer and each are diagnosed and treated in a particular way, each with their own influences on a GP’s decision to refer and associated costs to waiting [38]. For some types of cancer, such as lung cancer, there is a high time cost to waiting as the disease can be fast spreading and late diagnosis significantly reduces the chance of survival. Other forms of cancer are much more slow moving [11]. Therefore, there could be different responses by different cancer types which could be cancelling each other out. For example, the strong negative effect for some cancers may be diminished by null results for other cancers. We were unable to examine this relationship by cancer type as we do not have data on two-week wait referrals by cancer type, to be able to weight local hospital breaches of the two-week wait target by cancer type.

### Findings in relation to other studies

Studies which look at the effect of waiting times on patient demand in an elective setting [5–7], and on GP demand and on GP demand in an elective setting [8, 9], find negative elasticities of demand with respect to waiting times. While the magnitude of these elasticities tends to be low, we found no responsiveness of demand to waiting times in the setting we examine.

One previous study has examined the responsiveness of patient demand in an urgent setting [10], which is arguably more comparable to urgent diagnosis of suspected cancer. The study only focused on low-urgency patients in an urgent setting, but it too found a negative elasticity of demand with respect to waiting times.

Our results may differ from previous studies because a GP’s decision to refer urgently for a suspected cancer patient differs from other referrals in a number of ways. Firstly, GPs in the NHS in England considering referrals for suspected cancer are operating in a more tightly managed and resource-constrained system than those dominated by private health insurance.

Secondly, compared to waiting times elsewhere in the NHS, the two-week wait target for urgent cancer referrals is relatively short. This could mean that GPs are less responsive to longer waiting times for a diagnosis via the urgent referral route, as a delay in waiting times is likely to be in terms of days and weeks rather than months.

Lastly, anxiety around cancer is very high compared to other conditions [21]. Individuals fear the physical and emotional impact of a cancer diagnosis, compounded by the unpredictable nature of the disease [22, 23]. Increased anxiety could impact a patient’s benefit from referral, and could also increase the cost to a patient of referral if a patient who does not have cancer suffers increased anxiety and stress as a result of being unnecessarily subjected to the urgent referral process. The additional costs of a cancer referral to the patient may be internalised in the GP’s decision and reduce responsiveness to changes in waiting times for patients at the margin, even where diagnosis is most unclear.

### Implications for policymakers and future research

Policymakers should be aware that, in the case of urgent diagnosis for suspected cancer, GPs do not appear to respond to changes in local waiting times. This suggests that increasing capacity for urgent diagnosis of cancer via the two-week wait pathway could be successful in reducing waiting times. As the NHS in the UK currently faces record highs in waiting times in the aftermath of Covid-19 response delays and cancellations [39], this finding could have important implications for scaling supply capacity. While some supply constraints are likely to remain in place for some time, for example the NHS’s chronic and increasing workforce shortage [39] there might be shorter-term opportunities for scaling capacity including diagnostic imaging and technology changes which might increase productivity of these diagnostic opportunities still further.

Future research should aim to exploit exogenous sources of variation in waiting times to further confirm this relationship. While we have outlined plausible mechanisms for the lack of demand response, these could also be empirically explored in future research, for example with qualitative methods, to shed additional more light on the specific implications. Future work could also explore the heterogeneity of this effect by different cancer types, as diagnosis and treatment will vary significantly by type of cancer.

## Conclusions

The data suggest that volumes of urgent referrals made by GP practices are positively correlated with local waiting times, but we show this association is driven by differences between practices. This effect disappears when we focus on changes over time, suggesting that GP demand does not respond to waiting times for urgent suspected cancer diagnosis. Policymakers should consider increasing capacity for urgent diagnosis of cancer via the two-week wait pathway to reduce waiting lists in this setting.

## Supporting information

S1 Checklist PLOS ONE clinical studies checklist. (DOCX)

S1 Appendix Percentage of tumours diagnosed by each route in 2016, to the nearest 1%. (DOCX)

S2 Appendix Poisson regressions of the relationship between local hospital waiting times and GP demand. (DOCX)

S3 Appendix Inverse hyperbolic sine transformed linear regressions of the relationship between local hospital waiting times and GP demand. (DOCX)

S4 Appendix Relationship between GP demand and local hospital waiting times using a balanced panel. (DOCX)

S5 Appendix Unadjusted OLS estimates of the relationship between local hospital waiting times and GP demand. (DOCX)

S6 Appendix Practice-level descriptive statistics for patient outcomes. (DOCX)

S7 Appendix STROBE statement—Checklist of items that should be included in reports of observational studies. (DOCX)
