# Supplementary material for: The effect of local hospital waiting times on GP referrals for suspected cancer
Source: PLoS One. 2024 May 8;19(5):e0294061. doi: 10.1371/journal.pone.0294061 (PMC11078401; doi:10.1371/journal.pone.0294061)
Supplement: S1 Appendix — (DOCX) [file pone.0294061.s002.docx]

S1 Appendix: Percentage of tumours diagnosed by each route in 2016, to the nearest 1% [16]

1. Screening (5%)

- Cancers which are detected via a screening programme.

1. Two-Week Wait (38%)
   - Urgent GP referrals with a suspicion of cancer to see a hospital specialist
2. GP referral (24%)
   - Other routine and urgent GP referrals where the patient was not specifically referred via a Two Week Wait referral
3. Other outpatient (9%)
   - An elective route which begins with an outpatient appointment not referred by a GP (consultant to consultant referral, other referral, self-referral, dental referral or unknown referral)
4. Inpatient elective (2%)
   - Prior to diagnosis in an inpatient setting, there is no earlier information found prior to admission from a waiting list, booked or planned
5. Emergency presentation (19%)
   - Cancer which presents as an emergency (A&E, emergency GP referral, emergency consultant outpatient referral, emergency transfer, emergency admission or emergency attendance)
6. Death certificate only (0%)
   - Diagnosis is only made by death certificate
7. Unknown (3%)
   - There is no data available from inpatient or outpatient Hospital Episode Statistics (HES) or from cancer waiting times or screening
