# Supplementary material for: The effect of local hospital waiting times on GP referrals for suspected cancer
Source: PLoS One. 2024 May 8;19(5):e0294061. doi: 10.1371/journal.pone.0294061 (PMC11078401; doi:10.1371/journal.pone.0294061)
Supplement: S2 Appendix — (DOCX) [file pone.0294061.s003.docx]

S2 Appendix: Poisson regressions of the relationship between local hospital waiting times and GP demand

|  | Urgent referrals | | |
| --- | --- | --- | --- |
|  | Pooled | Between effects | Fixed effects |
|  |  |  |  |
| Local hospital breaches as a proportion of total treated | 1.446*** | 2.778*** | 1.059 |
|  | (0.129) | (0.772) | (0.0672) |
|  |  |  |  |
| Proportion aged 65+ years | 2.586*** | 1.790*** | 0.418*** |
|  | (0.128) | (0.243) | (0.0980) |
|  |  |  |  |
| Proportion aged under 18 years | 0.801*** | 0.821 | 0.570** |
|  | (0.0575) | (0.133) | (0.134) |
|  |  |  |  |
| Total QOF points achieved (proportion) | 1.168*** | 1.222** | 1.050 |
|  | (0.0429) | (0.121) | (0.0318) |
|  |  |  |  |
| Working status - Unemployed | 0.824*** | 0.542*** | 0.975 |
|  | (0.0482) | (0.109) | (0.0340) |
|  |  |  |  |
| Proportion reporting good overall experience of making appointment | 1.119*** | 1.211** | 1.008 |
|  | (0.0330) | (0.102) | (0.0210) |
|  |  |  |  |
| Proportion with a long-standing health condition | 1.325*** | 2.037*** | 0.974 |
|  | (0.0419) | (0.221) | (0.0175) |
|  |  |  |  |
| Proportion satisfied with phone access | 0.846*** | 0.811*** | 1.003 |
|  | (0.0184) | (0.0475) | (0.0211) |
|  |  |  |  |
| N*T | 37556 | 6,667 | 37527 |
| GP practice fixed effects | NO | NO | YES |
| GP practice random effects | NO | NO | NO |
| Year fixed effects | YES | NO | YES |

Notes: exposure variable is practice list size

Robust standard errors in parentheses. * p<0.10, ** p<0.05, *** p<0.01. Coefficients are Incident Rate Ratios.
