# Supplementary material for: The effect of local hospital waiting times on GP referrals for suspected cancer
Source: PLoS One. 2024 May 8;19(5):e0294061. doi: 10.1371/journal.pone.0294061 (PMC11078401; doi:10.1371/journal.pone.0294061)
Supplement: S3 Appendix — (DOCX) [file pone.0294061.s004.docx]

S3 Appendix: Inverse hyperbolic sine transformed linear regressions of the relationship between local hospital waiting times and GP demand

|  | Urgent referrals as a proportion of practice list size | | |
| --- | --- | --- | --- |
|  | Pooled | Between effects | Fixed effects |
|  |  |  |  |
| Local hospital breaches as a proportion of total treated | 0.0109*** | 0.0285*** | 0.00288 |
|  | (0.00252) | (0.00772) | (0.00184) |
|  |  |  |  |
| Proportion aged 65+ years | 0.0309*** | 0.0201*** | -0.00316 |
|  | (0.00124) | (0.00318) | (0.00705) |
|  |  |  |  |
| Proportion aged under 18 years | -0.00970*** | -0.00906*** | -0.00206 |
|  | (0.00163) | (0.00341) | (0.00650) |
|  |  |  |  |
| Total QOF points achieved (proportion) | 0.00542*** | 0.00535 | 0.00176 |
|  | (0.00121) | (0.00344) | (0.00119) |
|  |  |  |  |
| Working status - Unemployed | -0.00641*** | -0.0184*** | -0.000458 |
|  | (0.00135) | (0.00397) | (0.000944) |
|  |  |  |  |
| Proportion reporting good overall experience of making appointment | 0.00432*** | 0.00792*** | -0.0000917 |
|  | (0.000937) | (0.00268) | (0.000740) |
|  |  |  |  |
| Proportion with a long-standing health condition | 0.00850*** | 0.0208*** | -0.000307 |
|  | (0.000908) | (0.00291) | (0.000585) |
|  |  |  |  |
| Proportion satisfied with phone access | -0.00616*** | -0.00848*** | -0.00000232 |
|  | (0.000704) | (0.00192) | (0.000715) |
|  |  |  |  |
| Adjusted R^2^ | 0.179 | 0.0839 | 0.437 |
| N*T | 37531 | 6,667 | 37531 |
| GP practice fixed effects | NO | NO | YES |
| Robust standard errors | YES | NO | YES |
| Year fixed effects | YES | NO | YES |

Notes: all variables are inverse hyperbolic sine transformed.

Standard errors in parentheses. * p<0.10, ** p<0.05, *** p<0.01.
