# Supplementary material for: The effect of local hospital waiting times on GP referrals for suspected cancer
Source: PLoS One. 2024 May 8;19(5):e0294061. doi: 10.1371/journal.pone.0294061 (PMC11078401; doi:10.1371/journal.pone.0294061)
Supplement: S4 Appendix — (DOCX) [file pone.0294061.s005.docx]

S4 Appendix: Relationship between GP demand and local hospital waiting times using a balanced panel

|  | Volume of urgent referrals | | |
| --- | --- | --- | --- |
|  | Pooled | Between effects | Fixed effects |
|  |  |  |  |
| Target breaches as proportion of referrals | 0.436*** | 1.210*** | 0.0963 |
|  | (0.104) | (0.332) | (0.0741) |
|  |  |  |  |
| Proportion aged 65+ years | 1.200*** | 0.745*** | -1.136*** |
|  | (0.0559) | (0.137) | (0.255) |
|  |  |  |  |
| Proportion aged under 18 years | -0.244*** | -0.152 | -0.608** |
|  | (0.0791) | (0.146) | (0.255) |
|  |  |  |  |
| Total QOF points achieved (proportion) | 0.232*** | 0.304** | 0.0849* |
|  | (0.0540) | (0.149) | (0.0458) |
|  |  |  |  |
| Working status - Unemployed | -0.313*** | -0.836*** | -0.0590 |
|  | (0.0637) | (0.170) | (0.0411) |
|  |  |  |  |
| Proportion reporting good overall experience of making appointment | 0.172*** | 0.267** | -0.0186 |
|  | (0.0399) | (0.116) | (0.0313) |
|  |  |  |  |
| Proportion with a long-standing health condition | 0.388*** | 0.968*** | -0.0387 |
|  | (0.0405) | (0.125) | (0.0240) |
|  |  |  |  |
| Proportion satisfied with phone access | -0.169*** | -0.218*** | 0.0257 |
|  | (0.0298) | (0.0829) | (0.0288) |
|  |  |  |  |
| Practice list size | 1.160*** | 1.162*** | 0.864*** |
|  | (0.00480) | (0.00878) | (0.0230) |
|  |  |  |  |
| Adjusted R2 | 0.736 | 0.756 | 0.580 |
| N*T | 37,472 | 6,633 | 37,472 |
| GP practice fixed effects | NO | NO | YES |
| Robust standard errors | YES | NO | YES |
| Year fixed effects | YES | NO | YES |

Notes: all variables are inverse hyperbolic sine transformed.

Standard errors in parentheses. * p<0.10, ** p<0.05, *** p<0.01.
