# Supplementary material for: The effect of local hospital waiting times on GP referrals for suspected cancer
Source: PLoS One. 2024 May 8;19(5):e0294061. doi: 10.1371/journal.pone.0294061 (PMC11078401; doi:10.1371/journal.pone.0294061)
Supplement: S5 Appendix — (DOCX) [file pone.0294061.s006.docx]

S5 Appendix: Unadjusted OLS estimates of the relationship between local hospital waiting times and GP demand

|  | Urgent referrals | | |
| --- | --- | --- | --- |
|  | Pooled | Between effects | Fixed effects |
|  |  |  |  |
| Local hospital breaches as a proportion of total treated | 0.0656 | 0.475 | 0.111 |
|  | (0.195) | (0.667) | (0.0816) |
|  |  |  |  |
|  |  |  |  |
| Adjusted R^2^ | 0.0544 | -0.0000737 | 0.509 |
| N*T | 37556 | 6,667 | 37556 |
| GP practice fixed effects | NO | NO | YES |
| GP practice random effects | NO | NO | NO |
| Year fixed effects | YES | NO | YES |

Notes: exposure variable is practice list size

Robust standard errors in parentheses. * p<0.10, ** p<0.05, *** p<0.01. Coefficients are Incident Rate Ratios.
