# Supplementary material for: The effect of local hospital waiting times on GP referrals for suspected cancer
Source: PLoS One. 2024 May 8;19(5):e0294061. doi: 10.1371/journal.pone.0294061 (PMC11078401; doi:10.1371/journal.pone.0294061)
Supplement: S6 Appendix — (DOCX) [file pone.0294061.s007.docx]

S6 Appendix: Practice-level descriptive statistics for patient outcomes

|  | Mean | SD | Min | Max | Obs |
| --- | --- | --- | --- | --- | --- |
|  |  |  |  |  |  |
| *Patient outcomes* |  |  |  |  |  |
| Count of diagnoses via urgent referral | 17.813 | 14.032 | 0.000 | 162.000 | 37517 |
| Count of diagnoses via emergency presentation | 7.029 | 5.328 | 0.000 | 71.000 | 37517 |
| Count of diagnoses | 40.778 | 28.135 | 0.000 | 347.000 | 37517 |
|  |  |  |  |  |  |
| *Waiting times* |  |  |  |  |  |
| Local hospital breaches as a proportion of total patients referred | 0.052 | 0.023 | 0.000 | 0.225 | 37517 |
|  |  |  |  |  |  |
| *Covariates* |  |  |  |  |  |
| Registered population size | 7895.430 | 4632.929 | 1001.000 | 72227.000 | 37517 |
| Proportion aged 65+ years | 0.168 | 0.066 | 0.000 | 0.929 | 37517 |
| Proportion aged under 18 years | 0.209 | 0.042 | 0.000 | 0.539 | 37517 |
| Total QOF points achieved (proportion) | 0.959 | 0.058 | 0.161 | 1.000 | 37517 |
| Working status - Unemployed | 0.055 | 0.049 | 0.000 | 0.654 | 37517 |
| Proportion reporting good overall experience of making appointment | 0.743 | 0.136 | 0.111 | 1.000 | 37517 |
| Proportion with a long-standing health condition | 0.531 | 0.081 | 0.083 | 1.000 | 37517 |
| Proportion satisfied with phone access | 0.753 | 0.175 | 0.074 | 1.000 | 37517 |
|  |  |  |  |  |  |
